# Mapping of Novel QTL Regulating Grain Shattering Using Doubled Haploid Population in Rice (*Oryza sativa* L.)

**DOI:** 10.1155/2016/2128010

**Published:** 2016-06-22

**Authors:** Gyu-Ho Lee, In-Kyu Kang, Kyung-Min Kim

**Affiliations:** ^1^Division of Plant Biosciences, School of Applied Biosciences, College of Agriculture & Life Science, Kyungpook National University, Daegu 702-701, Republic of Korea; ^2^Department of Horticultural Science, College of Agriculture and Life Sciences, Kyungpook National University, Daegu 702-701, Republic of Korea

## Abstract

The critical evolutionary step during domestication of major cereals was elimination of seed shattering because the easy-to-shatter trait in wild relatives results in a severe reduction in yield. In this study, we analyzed the QTLs associated with shattering employing a high-density genetic map in doubled haploid (DH) population of rice (*Oryza sativa* L.). A genetic linkage map was generated with 217 microsatellite markers spanning 2082.4 cM and covering 12 rice chromosomes with an average interval of 9.6 cM between markers based on 120 DHLs derived from a cross between Cheongcheong indica type cultivar and Nagdong japonica type cultivar. In the QTL analysis, five QTLs pertaining to the breaking tensile strength (BTS) were detected in 2013 and 2015. Two regions of the QTLs related to BTS on chromosome 1 and chromosome 6 were detected. Several important genes are distributed in 1 Mbp region of the QTL on chromosome 6 and they are related to the formation of abscission layer. We decide to name this QTL* qSh6* and the candidate genes in the* qSh6* region can be employed usefully in further research for cloning.

## 1. Introduction

Major cereals were first cultivated approximately 10,000 years ago, when several plant characteristics, such as adaptation of flowering time to regional areas, increase in the number of seeds, variation in plant architecture and seed shape, response to increased fertility, and prevention of seed shattering, were altered by selection. These efforts of selection have generated a wide variety of crops [1, 2]. Rice, which is the largest food crop cultivated worldwide, meets the caloric needs of millions of people and is used as a staple food by half of the world's population. There are two distinct types of domesticated rice,* Oryza sativa* or Asian rice and* Oryza glaberrima* or African rice; each has unique domestication histories [3]. The critical evolutionary step during domestication of major cereals was elimination of seed shattering because the easy-to-shatter trait in wild relatives results in a severe reduction in yield [1, 3] and wild rice also disperses seeds freely at maturity to guarantee the propagation, while cultivated rice retains seeds on the straw to make harvest easy and decrease the loss of production [4]. The degree of shattering can be distinguished into several types, easy-shattering, moderate shattering, and hard or nonshattering. The type of shattering is important to consider when selecting combine machines to prevent loss of yield during harvest. Specifically, small head-feeding combines are usually employed to harvest hard or nonshattering varieties, while large combine harvester-threshers are used for the moderate shattering varieties. Therefore, measurement of grain shattering is critical to enable the best harvesting method and reduce yield loss [5]. A digital force gauge to measure the degree of shattering is useful [6–10]. The device can measure the greatest amount of force against the direction of pulling during removal of seeds from a plant (Kg/pull). In the different methods, the shattering trait can be measured as the percentage of fallen spikelets among total spikelets of each panicle [11, 12]. The bagging panicles method is similar method to evaluate grain shattering for scoring the proportion of seeds naturally shed, shed by hand, and not shed by hand as 2, 1, and 0, respectively [13]. Also shattering habit was evaluated in shattering scale by hand gripping and recorded it on 1−9 scale [14]. However, there are several inevitable factors such as water content, growth stage, and man-made errors.

The shattering habit of rice is considered to be under relatively simple genetic control when compared with other characteristics related to domestication. Several recessive genes associated with the formation of an abscission layer,* sh2*,* sh4,* and* sh-h* on chromosomes 1, 3, and 7, have been reported [5, 15, 16].* Sh3* and* SHA1* on chromosome 4 from wild rice were also reported [3, 17]. The grain shattering is considered to be caused by abscission layer [5]. The morphology of the abscission layer can differ in many rice varieties that show varying degrees of shattering [18–20]. Accordingly, it is important to elucidate the molecular mechanism to determine why some varieties do not have abscission layers and have an easy-shattering trait.

Various segregation patterns ranging from being monogenic to multigenic and depending on the crosses have been found, indicating that the shattering degree of cereals is a complex trait influenced by many genetic and environmental factors [21]. Many genetic studies for the seed shattering habits have been carried out using populations of natural variation from a cross between* indica* variety with shattering type and* japonica* variety with nonshattering type [2, 6, 11, 13, 14, 22]. A major QTL of grain shattering in rice,* qSH1*, was revealed to encode a BEL1-type homeobox gene [1]. A single-nucleotide polymorphism in the 5′ regulatory region of the* qSH1* gene has also been shown to lead to the loss of seed shattering because of the absence of an abscission layer formation. In this study, we tried to detect QTLs associated with shattering traits with candidate genes by development of a genetic linkage map using 122 DHLs derived from a cross between the* indica* cultivar Cheongcheong and* japonica* cultivar Nagdong.

## 2. Materials and Methods

### 2.1. Plant Materials

The DH population for construction of the genetic map was developed by anther culture of the F_1_ derived from a cross between Cheongcheong and Nagdong. Cheongcheong is a Tongil type rice variety that has resistance to the brown plant hopper, while Nagdong is the main variety cultivated in the regional area and has a partial abscission layer on the pedicel tissues [10]. The CNDH population was cultivated in a paddy field in 2013 and 2015 in the experimental fields of Kyungpook National University at Gunwie in Korea.

### 2.2. DNA Isolation and Genotyping

The genomic DNA of the parents cultivars and 120 DHLs were isolated from fresh leaves by CTAB method. In total, 217 microsatellite markers were used to determine genotype of the population for construction of a genetic linkage map. A total of 24 *μ*L consisted of 2 *μ*L of 10–20 ng/*μ*L template DNA, 1 *μ*L of 5–10 pM of each primer, 0.1 *μ*L of* Taq* polymerase (Imclone Biotech Co., IN5001), 0.375 *μ*L of dNTPs mixture, 2.4 *μ*L of 10x Ex buffer, and 17.125 *μ*L of nuclease-free water (QIAGEN, category number 129114). Analyses were conducted using GeneAmp PCR System 2700 (Applied Biosystems, USA). The reaction consisted of initial denaturation for 5 minutes at 96°C followed by 34 cycles of 30 seconds at 96°C, 30 seconds at 55°C, and 1 minute at 72°C, followed by final extension for 8 minutes at 72°C and then storage at 4°C PCR. Finally, the PCR products were confirmed by electrophoresis (QIAGEN, QIAxcel) for genotyping.

### 2.3. Construction of a Genetic Map

The data derived from PCR genotyping were input into Mapmaker 3.0 to calculate the genetic distance by applying the Kosambi function. A total of 217 microsatellite markers were used to generate a genetic linkage map spanning the 12 rice chromosomes using Mapmaker/EXP 3.0. The microsatellite markers were provided by the National Institute of Crop Science (NICS), RDA. The linkage among markers was determined by the group command at an LOD threshold of 3.0 and a recombination fraction of 0.30.

### 2.4. Phenotype Evaluation and QTL Analysis

**Figure 1:**
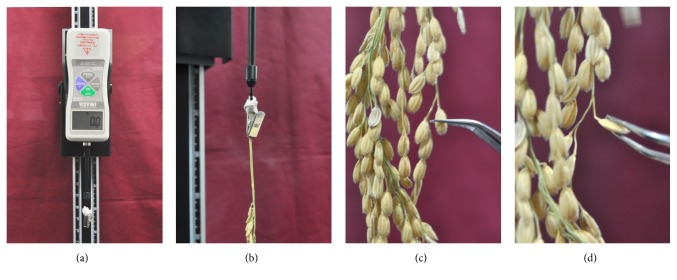
Measurement of seed shattering degree using a force gauge. (a) The device used to measure shattering degree was fixed to a stand reversely. (b) A holder was used to link tightly with a panicle stem. (c, d) The breaking tensile strength (BTS) was measured by bending and pulling a spikelet.

The panicles samples were harvested at 40 days after heading (DAH) and then placed at room temperature for a week in 2013 while all the lines of the population were harvested at 40 DAH and placed at room temperature for three days in 2015. Five panicles per line were harvested for the measurement and shattering degree of spikelet was investigated as breaking tensile strength. To increase the precision and consistency, the shattering degree of spikelet was measured using a digital force gauge (Imada, Japan). The panicles were fixed to the force gauge to measure breaking tensile strength by pulling and bending grain spikelet using forceps (Figure 1). In total, 50 grains with 5 pedicles were removed by pulling and bending randomly. The Windows QTL cartographer 2.5 was used for analysis of QTLs associated with grain shattering. Composite interval mapping (CIM) was conducted for the entire genome by WinQTLCart 2.5 at a threshold of LOD 2.5 [23] after input of all required data and the heritability is set for calculation at 0.8 as a default value by the program. QTLs detection was attempted three times according to the date of harvest.

## 3. Results

### 3.1. Construction of the Genetic Map

Total markers consisting of 217 microsatellites were used to construct a genetic linkage map of the CNDH population derived from a cross between the* indica *cultivar, Cheongcheong, and* japonica* cultivar, Nagdong. The genetic linkage map of 12 chromosomes is generated by Mapmaker 3.0 covering 2082.4 cM in the entire genome with an average of 9.6 cM between markers (Supplemental Figure  S1) (see Supplementary Material available online at http://dx.doi.org/10.1155/2016/2128010).

### 3.2. Phenotype Evaluation

The bending and pulling strength of the DH population at 40 days after heading (DAH) in 2013 and 2015 were measured by unit of gravity force (gf) using a digital force gauge. The determination of shattering degree in 2013 and 2015 has shown that the shattering value of Cheongcheong is of easy-shattering type and Nagdong is of moderately difficult-shattering type (Figure 1). The two different breaking tensile strengths (BTSs) by pulling and bending have a correlation over 87% with significance at the level of 0.01 (Table 2, Supplemental Figure  S3). In addition, the BTS has shown the other grain characteristics including grain width, thickness, and thousand grain weight are related to the BTS below 30% with 0.05 of significance level (Table 2). The pulling strength in 2015 only was similar to normal distribution.

### 3.3. Analysis of QTLs

A total of 3 QTLs for pulling strength and 4 QTLs for bending were confirmed on chromosomes 1, 4, 6, 9, and 10 in 2013 while 3 QTLs for pulling strength on chromosomes 1, 2, 6, and 4 QTLs for bending strength were detected during 2015 (Table 2, Figure 3). The qPS1 was represented as 5.81 as the highest LOD value with 11% of phenotypic variation (*R* ^2^) in 2013 while 6.93 of LOD for qPS6 with 13% of phenotypic variation was the highest in 2015. The qPS1 and qPS10 were detected in the same region of qBS1 on RM11966–RM11849 and qBS10 on RM25219–RM25036 in 2013. The QTLs confirmed on chromosomes 1 (qPS1) and 6 (qPS6) also were placed on regions, RM11966–RM11849, and RM20632–RM439 in 2015, respectively. The position of qPS6 in 2015 was equal to qPS6 in 2013 and the QTLs for pulling and bending strength with around 3.7 of LOD and 0.08% of phenotypic variation on chromosome 10 were detected on the same region, RM25219–RM25181, in 2013.

## 4. Discussion

**Figure 2:**
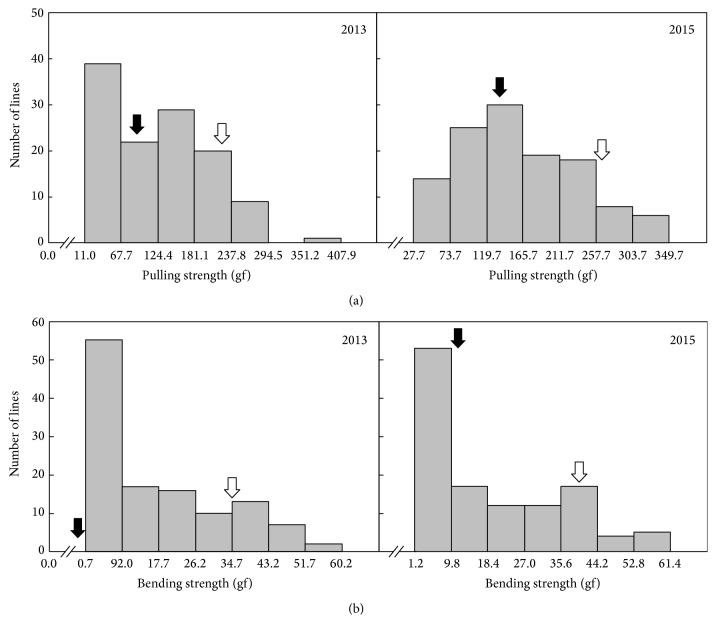
The frequency distribution for BTS in DH population. BTS was measured as pulling strength (a) and bending strength (b) for two years. The black arrows are represented as Cheongcheong while the white arrow indicates Nagdong.

**Figure 3:**
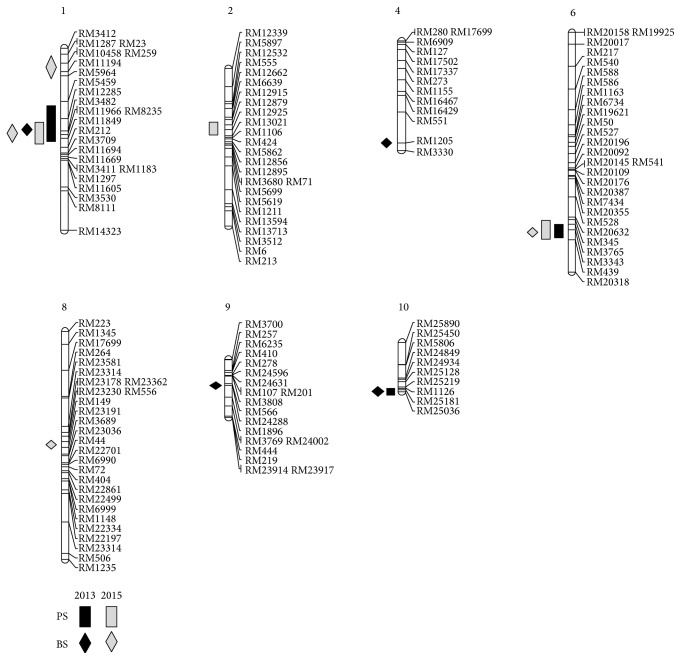
The chromosomal locations of the QTLs related to pulling strength (PS) and bending strength (BS) in 2013 and 2015. The rectangles and diamonds are the QTL associated with pulling and bending strength.

Grain shattering is considered to be an important trait because it can affect patterns of harvest using combine machine and yield [5]. In this study, 217 microsatellite markers were selected from 1402 microsatellite markers by polymorphism test for construction of a genetic linkage map and it was employed to analyze QTLs associated with grain shattering. The grain shattering can be measured in different methods. The bagging method is to obtain the ratio of grains naturally shed because the pedicels of rice are sensitive to shaking in harvest period. Therefore, the shattering was measured in the ratio of fallen spikelets in the entire spikelet of each panicle [11, 12]. It was also evaluated using two bagged panicles per plant and the proportion of seeds naturally shed, shed by hand gripping, and nonshed by hand gripping was recorded by scores 2, 1, and 0, respectively [13]. The shattering degree was measured by hand gripping and recorded on 1–9 scale [14]. However, many factors such as growth environment, growth stage, water content, and human errors can influence the shattering evaluations. Recently, the grain shattering has also been measured using a digital force gauge that can provide more precise data than other methods by pulling or bending grain pedicels [1, 5, 10]. In the shattering investigation, the parental cultivars of the DH population, Cheongcheong and Nadong, have shown that they are represented as of easy-shattering and moderately difficult shattering type, respectively (Table 1). However, the shattering degree of the parents is represented to be increased in 2015 (Figure 2). It can be assumed that it is due to the different drying time after harvest. There is a gap for three days between 2013 and 2015.

Many QTLs associated with shattering trait have been reported from the previous studies (Table 4). In this study, five QTLs were detected on chromosomes 1, 4, 6, 9, and 10 in 2013 and also five QTLs were identified on chromosomes 1, 2, 6, and 8 in 2015. The QTLs associated with pulling strength and bending strength on chromosome 1 were named qPS1 and qBS1 according to the QTL nomenclature [4, 24]. However, the qPS1 and qBS1 detected in 2013 and 2015 have shared the region of* qSH1* on chromosome 1 with 68.6% of phenotypic variation [1]. The* SHA1* on chromosome 4 can be considered to be a major gene related to grain shattering and also the QTL for bending strength on chromosome 4 (qBS4) has been detected on the opposite region of chromosome 4 compared with* SHA1*. However, there was only one QTL related to BTS showing relatively low LOD value among the detected QTLs in the present study. Therefore, we tried to focus on other QTLs for grain shattering newly found on chromosome 6 (qPS6) with the highest LOD value in 2015 (Table 3). Although two QTLs,* sh6* and* qSH6-1*, related to grain shattering on chromosome 6 were found in other researches [9, 14], the QTLs on chromosome 6 in current study have been detected newly at different position. The qPS6 was detected from the QTL analysis in 2013 and 2015 and the regions of qPS6 and qBS6 were same location flanked by RM20632–RM439. The correlation analysis has shown that pulling and bending strength have over 80% of highly significant correlation at the level of 0.01 (Table 2). Thus, the two QTLs, the qPS6 and qBS6, were changed in the equal name of* qSh6* and we tried to find candidate genes for* qSH6* distributed on the 1 Mbp region.

The pulling and bending strength of pedicel in shattering trait have a considerable relationship with the existence of abscission layer [1, 5]. The abscission layer of pedicel is essential for seed propagation and it is well known that it is affected by many factors such as ethylene [25] and injury due to insect or fungal attack [26]. Therefore, Os06g6991100 encoding AP2 superfamily and describing ethylene responsive factor 121 (ERF121) and APETALA2/ethylene-responsive element binding protein 61 (AP2/EREBP61) is considered as one of the important candidate genes. The* SHAT1* gene, which encodes APETALA2 transcription factor on chromosome 4, is required for seed shattering through specifying abscission zone development in rice [27]. Also the existence of many protein kinase-related and pathogen-related genes indicates that the shattering habit is affected by signaling, which can change in hormone, from many environmental factors [27]. Os06g069700 and Os06g0700700 encode heavy metal ATPase 1 (HMA1) and heavy metal ATPase 2 (HMA2). They are related to transport heavy metal, cadmium, zinc, and copper. The high accumulation of cadmium rice for phytoremediation indicated that cultivars can be developed with resistance in shattering and lodging by marker-assisted selection [28]. Os06g0701600 and Os06g0701700 that encode high-affinity K^+^ transporter 2;4 (HKT2;4) and HKT1 are related to control transportation of cNa^+^, K^+^, and Ca^2+^. The HKT rice gene, named* OsHKT2*;*4,* can function as both ion carriers and channels [29]. Calcium is one of the components of cell wall and known to maintain both cell wall and membrane in the abscission process. In particular the calcium pectate functions as the cementing substance between cells in the bean leaf [30, 31]. Furthermore, calcium can affect inhibiting the formation of abscission layer [32]. These studies indicate that* OsHKT2*;*4* can affect the formation of abscission layer indirectly. The Os06g0701900 and Os06g0702000 encode small auxin-up RNA (SAUR). The exact function of SAUR is unknown but some of them play a role in auxin signaling with calcium and calmodulin [33]. Therefore, we can conclude that some of candidate genes distributed on* qSh6* are involved in formation of abscission layer and recognition of ethylene directly or indirectly by many complex signalings. Among the 165 candidate genes that can be searched in the* qSh6* region with 1 Mpb, some genes are considered as important in shattering habit. The present study will be useful in the separation and introduction of the genes to the parental cultivars for confirmation of gene function. We can presume the influence of the genes for grain shattering habit by previous researches indirectly. Thus, the comparison between expression of the genes and phenotype via gene introduction to parental cultivar after cloning the genes will be helpful to understand their function.

## 5. Conclusion

The shattering habit is really complex phenomenon for propagation of seed. We could find newly detected QTLs related to breaking tensile strength. Among the seven QTLs in 2015, the* qSh6* that explains 13% of the phenotypic variation with 6.93 of LOD will be exploited for cloning in further research. The* qSh6* was detected in QTL for pulling and bending strength region on chromosome 6 equally in 2015. About seven candidate genes describing ethylene response factor (ERF), heavy metal ATPase (HMA), high-affinity K+ transporter (HKT), and small auxin-up (SAUR) were found among the 165 gene loci in 1 Mbp region. They will be employed in further cloning research for introduction of the gene to the parental plant and for rice breeding.

## Supplementary Material

The correlation analysis was conducted by IBM SPSS Statistics 22.

## Figures and Tables

**Table 1:** Evaluation for shattering degree by force gauge.

Trial	2013		2015	
	PS (gf)^a^	BS (gf)	PS (gf)	BS (gf)
Cheongcheong	98.5 ± 31.0^b^	10.9 ± 3.5	157.4 ± 19.1	6.45 ± 4.05
Nagdong	219.7 ± 24.6	38.1 ± 2.9.	261.6 ± 16.5	34.7 ± 10.21
DH population	126.7 ± 79.2	18.9 ± 16.0	161.2 ± 74.6	17.0 ± 14.8

^a^Gravity force; ^b^mean ± standard deviation.

**Table 2:** Analysis of the correlation coefficients for BTS and grain characteristics.

Year	Trait	PS	BS	GL	GW	GT	RLW	TGW
2013	PS	1	0.882^*∗∗*^	−0.077	0.245^*∗∗*^	0.226^*∗*^	0.157	0.221^*∗*^
	BS		1	−0.146	0.285^*∗∗*^	0.228^*∗*^	−0.221	0.119^*∗*^
	GL			1	−0.444^*∗∗*^	−0.339^*∗∗*^	−0.861^*∗∗*^	0.368^*∗∗*^
	GW				1	0.784^*∗∗*^	−0.827^*∗∗*^	0.554^*∗∗*^
	GT					1	−0.633^*∗∗*^	0.600^*∗∗*^
	RLW						1	−0.076
	GW							1

2015	PS	1	0.872^*∗∗*^	−0.164	0.292^*∗∗*^	0.306^*∗∗*^	−0.231^*∗*^	0.203^*∗*^
	BS			−0.204^*∗*^	0.250^*∗∗*^	0.236^*∗*^	−0.245^*∗∗*^	0.116
	GL				−0.444^*∗∗*^	−0.339^*∗∗*^	0.861^*∗∗*^	0.368^*∗∗*^
	GW				1	0.784^*∗∗*^	−0.827^*∗∗*^	0.554^*∗∗*^
	GT					1	−0.633^*∗∗*^	0.600^*∗∗*^
	RLW						1	−0.0765
	TGW							1

^*∗*^Significant at the level of 0.05 and ^*∗∗*^significant at the level of 0.01. PS: pulling strength; BS: bending strength; GL: grain length; GW: grain width; GT: grain thickness; RLW: ratio of length/width; TGW: thousand grain weight.

**Table 3:** The QTL analysis related to pulling and bending strength in 2013 and 2015.

Year	Chr.^a^	Locus	LOD	Add.^b^	*R* ^2c^	Marker interval	Allele
2013	1	qPS1	5.81	29.57	0.11	RM11966-RM11849	Cheongcheong
	6	qPS6	4.25	24.64	0.08	RM20632-RM439	
	10	qPS10	3.73	22.80	0.07	RM25219-RM25181	
						
	1	qBS1	3.43	4.12	0.06	RM11966-RM11849	Cheongcheong
	4	qBS4	3.27	4.79	0.08	RM1205	
	9	qBS9	2.78	3.75	0.05	RM566-RM24288	
	10	qBS10	3.76	4.77	0.08	RM25219-RM25181	

2015	1	qPS1	5.14	26.15	0.11	RM11966-RM11849	Cheongcheong
	2	qPS2	4.68	23.29	0.09	RM12915-RM12925	
	6	qPS6	6.93	28.70	0.13	RM20632-RM439	
						
	1	qBS1-1	4.95	6.39	0.14	RM10458-RM11194	Cheongcheong
	1	qBS1-2	4.26	5.16	0.11	RM11966-RM11849	
	6	qBS6	3.13	4.33	0.07	RM20632-RM439	
	8	qBS8	3.03	4.69	0.08	RM149-RM23191	

^a^Chromosome, ^b^additive effect, and ^c^phenotypic variation.

**Table 4:** Comparison of QTL identified from previous studies.

Traits	Chr. number	Population type (size)	Marker (number)	Parental cultivars	QTL name	References
PNS	1, 4, 5, 8	F_2_ (172)	RFLP, SSR, AFLP (348)	Aijiao Nante (*indica*), P16 (*O. rufipogon*)	sh1, sh4, sh5, sh8	Xiong et al. (1999) [11]

PNS	1, 4, 8, 11	RILs (125)	RFLP, isozyme morphological (147)	Pei-Kuh (*indica*), W1944 (*O. rufipogon*)	qSHT-1, qSHT-4, qSHT-8, qSHT-11	Cai and Morishima (2000) [13]

PNS	1	DH (151)	SSR (68)	Miara (*japonica*), C6′ (*japonica*)	*Sh-2*	Bres-Patry et al. (2001) [12]

PNS	1, 3, 6	RIL (120)	SSR (124)	Hwayeongbyeo (*japonica*), W1944 (*O. rufipogon*)	*sh1, sh3, sh6*	Lee et al. (2005) [14]

PS	1, 2, 5, 11, 12	F_2_ (156)	RFLP, RAPD (609)	Kasalath (*indica*), Nipponbare (*japonica*)	*qSH1*	Konish et al. (2006) [1]

PS	3, 4, 8	F_2_ (304)	SSR	CL16 (*indica*), IRGC 80470 (*O. nivara*)	*sh3, sh4, sh8*	Li et al. (2006) [7]

PS, BS	7	F_2_ (240)	SSR (77)	Hsh (*japonica*), Blue & Gundil (*japonica*)	*sh-h*	Ji et al. (2006) [5]

SR	1, 3, 4, 11, 12	F_2_ (376)	SSR	Teqing (*indica*), YJCWR (*O. rufipogon*)	*SHA1*	Lin et al. (2007) [3]

PS	1, 6	CSSL (103)	SSR (132)	93-11 (*indica*), Nipponbare (*japonica*)	*qSH1-1*, *qSH6-1*	Zhu et al. (2009) [9]

SS, AL, PS, BS	1, 3, 4, 5, 8, 9	DH (120)	SSR, STS (182)	Samgang (*indica*), Nagdong (*japonica*)	Qss1, Qss3, Qss4, Qss5-5, Qal1, Qal5-1, Qps8, Qbs9	Qin et al. (2010) [10]

PS: pulling strength, BS: bending strength, PNS: percentage of natural shed, SR: shattering rate, SS: shattering scale, and AL: abscission layer.
