# Supplementary material for: Mapping of Novel QTL Regulating Grain Shattering Using Doubled Haploid Population in Rice (Oryza sativa L.)
Source: Int J Genomics. 2016 Jun 22;2016:2128010. doi: 10.1155/2016/2128010 (PMC4933857; doi:10.1155/2016/2128010)
Supplement: Supplementary file 1 — The correlation analysis was conducted by IBM SPSS Statistics 22. [file 2128010.f1.doc]

**Supplementary material**

**Analysis of shattering-related QTLs using a genetic map in rice (*Oryza sativa* L.)**

**
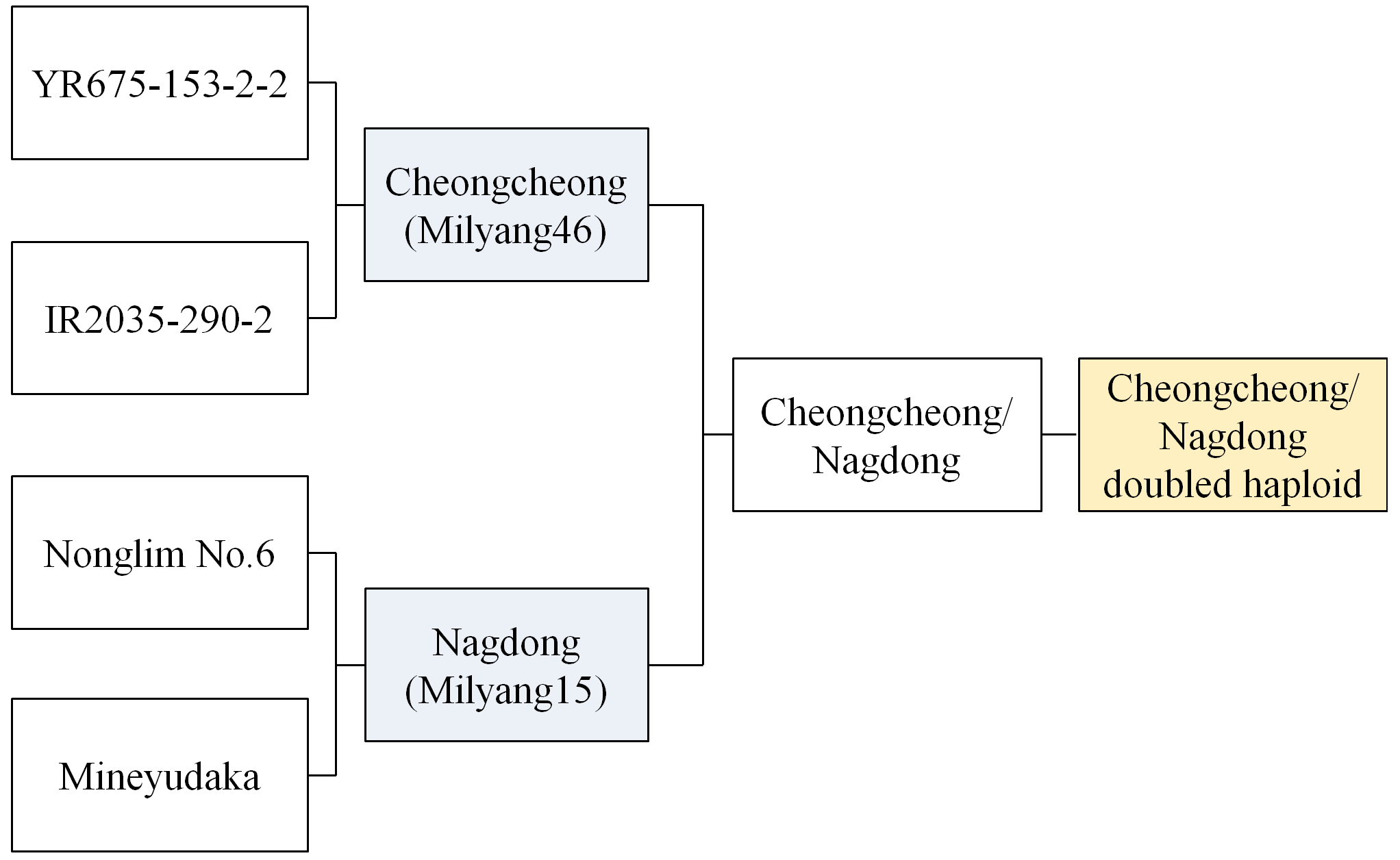
**

Supplemental Figure S1. The genealogy of CNDH population (yellow) and their parental varieties Cheongcheong and Nagdong (blue). The Cheongcheong is a Tongil-type cultivar from a cross *indica*/*japonica* and the Nagdong is *japonica* type cultivar.


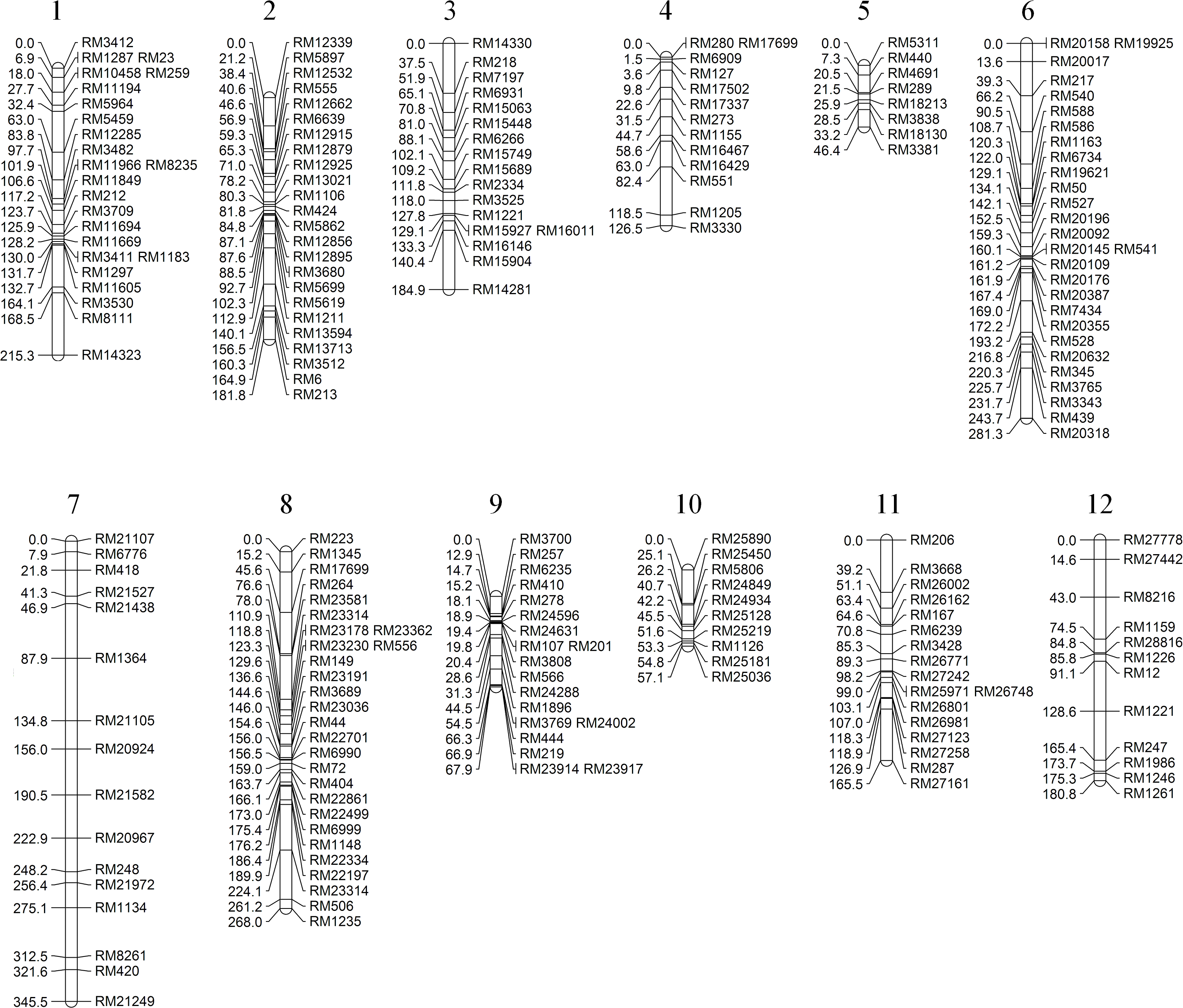


Supplemental Figure S2. Linkage map of CNDH population of rice from a cross of Cheongcheong and Nagdong. Left side of the chromosomes indicates genetic distance using centimorgan (cM) and the right side indicates SSR markers.


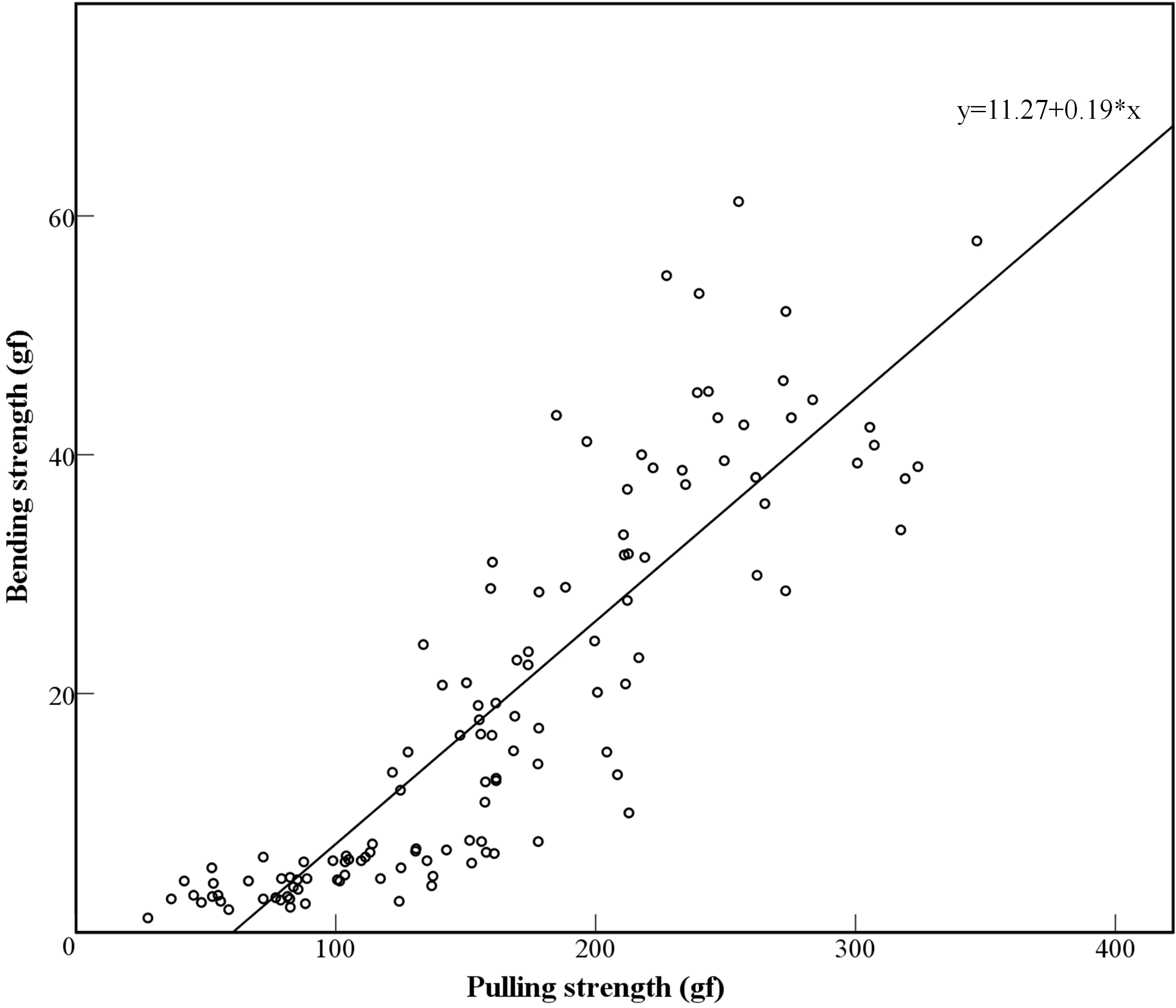


Supplemental Figure S3. The correlation analysis between pulling strength and bending strength.
